# Effect of Antioxidants and B-Group Vitamins on Risk of Infections in Patients with Type 2 Diabetes Mellitus

**DOI:** 10.3390/nu5030711

**Published:** 2013-03-05

**Authors:** Salah Gariballa, Bachar Afandi, Mamoon Abu Haltem, Javed Yassin, Awad Alessa

**Affiliations:** 1 Internal Medicine, Faculty of Medicine & Health Sciences, United Arab Emirates University, Al Ain, PO Box 17666, United Arab Emirates; E-Mails: javed.yasin@uaeu.ac.ae (J.Y.); a.alessa@uaeu.ac.ae (A.A.); 2 Tawam Hospital, Al Ain, PO Box 15258, United Arab Emirates; E-Mails: bachar.afandi@tawamhospital.ae (B.A.); mhaltem@tawamhospital.ae (M.A.H.)

**Keywords:** dietary supplement, risk of infection, type 2 diabetes mellitus, B-group vitamins, antioxidant vitamins

## Abstract

Previous studies have revealed that diabetic patients have a decline in immunity and an increased risk of infections, and this may be associated with poor micronutrient status. The aim of this study was to measure the effect of dietary supplements on risk of infection in patients with type 2 diabetes mellitus. One hundred patients with type 2 diabetes mellitus were randomly assigned to receive an oral dose of daily B-group vitamins and antioxidant vitamins (*n* = 50) or an identical placebo (*n* = 50) daily for 90 days. Patients had baseline, three and 12 month assessment for nutritional status, fruits and vegetables intake, physical activity and self-reported infections. Supplementation with antioxidants and B-group vitamins significantly increased the plasma concentration of vitamin E and folate and reduced homocysteine in the intervention group (*p-*values were 0.006, 0.001 and 0.657, respectively). The number of infections reported by the treatment group after three months of supplements was less than that reported by the placebo group, 9 (27%) *vs.* 15 (36%) (*p* = 0.623). Corresponding numbers of infections at 12 months were 25 (67.5%) and 27 (56.3%), respectively (*p* = 0.488). Up to 90% of the diabetic patients were either overweight or obese with a sedentary life style, and their body weight increased further during three months of follow up. The study showed that multivitamin supplements improved vitamin blood concentrations; however, this did not reduce the number of infections in diabetic patients.

## 1. Introduction

The United Arab Emirates (UAE) society has been through rapid socioeconomic and social changes with urbanization over the last 40 years. Accompanying changes in diet and lifestyle are therefore leading to a growing epidemic of overweight/obesity, type 2 diabetes and other related cardiovascular disease (CVD) [1]. As a result, the UAE has the second highest prevalence of diabetes mellitus in the world.

Patients with diabetes mellitus are considered to be more prone to infections than those without diabetes mellitus [2]. In animal and *in vitro* studies, the host’s immune functions were reported to be disturbed by short- or long-term hyperglycemia, including neutrophil bactericidal function [3], cellular immunity [4] and complement activation [5]. These defects in the immune system, along with vascular insufficiency, render diabetic patients at higher risk for a variety of severe or invasive infections, such as pyogenic bacterial infections, necrotizing infections, Candida infections or other fungi infections [6,7]. Although specific defects in innate and adaptive immune function have been identified in diabetic patients in a number of studies, the relevance of these findings to the increased risk of infections in diabetic patients remain unclear [8]. Furthermore defects in immunity might be one possible reason for this increased incidence of infections [4]. A number of studies have reported alteration in micronutrient status, notably ascorbic acid and B vitamins, and in some of these studies, deficiency of certain micronutrients have been associated with the presence of diabetic complications, including infections [9].

There is also extensive evidence of an association between the immune system and nutrient status. Poor nutrition increases susceptibility to infection, and infection in turn, has an adverse effect on nutritional status [10]. 

To our knowledge, there are no studies conducted in the UAE that examined the effect of dietary supplement on the risk of infection in patients with type 2 diabetes mellitus. This study aimed to study the effect of antioxidants and B-group vitamins on the risk of infections in a community of free living patients with type 2 diabetes mellitus. 

## 2. Experimental Section

### 2.1. Material and Methods

All patients with type 2 diabetes mellitus visiting the diabetes center at the Tawam hospital for regular follow up of their diabetes were considered for the study. Tawam hospital is one of the two main teaching hospitals in the city of Al Ain, serving a total population of 400,000. 

Patients aged 18 years and above with type 2 diabetes were approached and invited to take part in the study. Individuals with severe chronic clinical or psychiatric disease, participating in other intervention trials, on dietary supplements and those unable to give an informed written consent were excluded. The local research ethical committee has approved the study. 

Following informed written consent and their recruitment to the study, eligible patients had a fasting 10 mL of blood taken for measurements of antioxidants, B group vitamins and related nutritional and biochemical variables at baseline. Patients were then randomly assigned to receive a capsule of antioxidant vitamins (221 mg of α-tocopherol and 167 mg of vitamin C) and B-group vitamins (1.67 mg folic acid, 1.67 mg vitamin B-2, 20 mg vitamin B-6, 0.134 mg vitamin B-12) or an identical placebo daily for 90 days. Patients otherwise managed according to standard practice. Clinical assessment that included control of diabetes and associated risk factors and complications was also performed at baseline and repeated at 3 months. Fruits and vegetable intake, physical activity and rate of infections were assessed at 3 and 12 months post-randomization.

### 2.2. Supplements and Placebo

The Placebo tablet was identical to the active supplement vitamin capsule. No doctor, investigator, nurse or patient could differentiate between the active treatment capsule and the placebo capsule.

### 2.3. Compliance with Trial Medications

Compliance was assessed by counting the remaining supplement tablets and analysis of blood vitamin levels collected immediately after the end of the supplement period at the 3-month follow up visit.

### 2.4. Measurements

Anthropometric data, including body weight, height and body mass index (BMI), were measured using the Tanita body composition analyzer. Waist circumference was measured using a flexible plastic tape. 

#### 2.4.1. Measurement of Fruits and Vegetables Intake

An abbreviated semi-quantitative food frequency questionnaire designed for self-administration following a brief verbal discussion was used to assess subject’s fruit and vegetables intake. It specifies the usual frequency of consumption of food items during the previous 12 months and assesses the average weekly nutrient consumption of each individual. The full version of the questionnaire has been developed and validated against a 7-day weighted dietary intake. It has also been compared with numerous other diets and used in many other studies [11].

#### 2.4.2. Measurement of Physical Activity

A questionnaire was used to assess occupation and leisure-related physical activity. Data were obtained on frequency and duration of daily or weekly physical activity sessions for at least 20 min or more in which subjects became breathless or sweat. Questions were also asked about the number of hours subjects spend in bed (this included time spent reading, watching television or sleeping).

### 2.5. Infection Diary

Infection incidence data were obtained from symptoms and treatment checklist diaries recorded during fact-to-face interviews at 3 months and through telephone interviews at 12-month follow-up. Using the infection incidence diary record, the research officer then assigned a specific diagnosis and duration of illness. Standard criteria were used to diagnose common adult infectious illnesses (upper respiratory tract, lower respiratory tract infection, influenza-like illnesses, sore throat, sinusitis, skin infections, eye and ear infections, gastrointestinal infections and urinary tract infections [12].

### 2.6. Blood Samples

Fasting blood samples were drawn into 2 vacutainer tubes, containing potassium EDTA as anticoagulant. The samples were thoroughly mixed at room temperature and immediately transferred to the laboratory. Both tubes were centrifuged immediately for 10 min at 4000 rotations/min. Plasma and serum were collected and stored at −80°C for future determinations of vitamins. 

### 2.7. Statistics and Analysis

#### 2.7.1. Randomization Strategy

The randomization sequence was generated using computer generated tables, concealed in sequentially numbered sealed opaque envelopes and kept in a clerical office.

#### 2.7.2. Sample Size Calculation

A previous study has shown that supplementation of diet with 400 mg of vitamin C increased plasma vitamin C by 45% [13]. Therefore, a sample size of 96 diabetic patients (48 treatment and 48 controls) would allow the detection of a 30% difference between groups in total plasma vitamin C concentrations with 80% power and a type 1 error probability of ≤0.05.

#### 2.7.3. Analysis

A repeated measures analysis of variance (ANOVA) test was used to test within subject differences and a *p-*value < 0.05 was considered significant. Differences in cumulative changes between groups were adjusted for age, BMI, duration and treatment of diabetes. The Mann—Whitney *U* test was also used.

#### 2.7.4. Quality Assurance

To optimize accuracy of data collection, entry and analysis, two individuals performed quality assurance by independently checking the accuracy of data entry performed. A random audit of 20 cases of the entered data against the paper based forms was done twice by two different operators and found that more than 96% of the data entry process was accurate. 

## 3. Results

**Figure 1:**
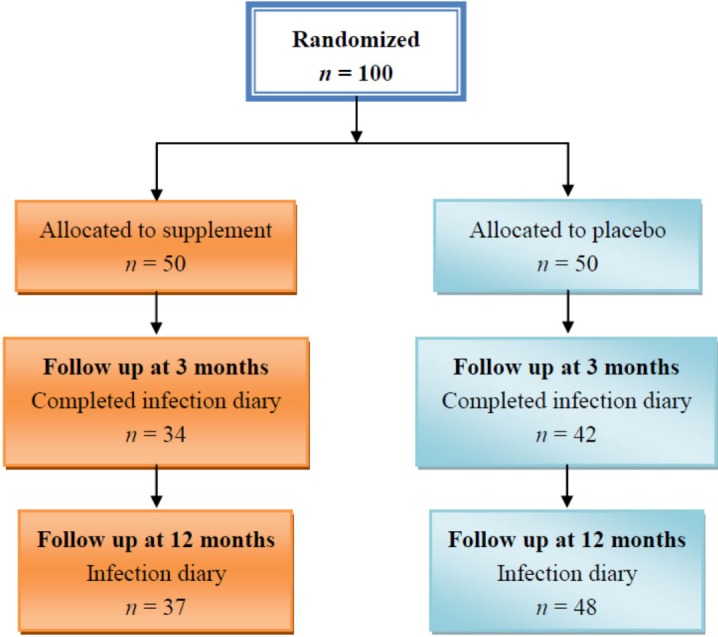
Enrolment, treatment and follow up of study patients.

One hundred diabetic patients were recruited to the study. Forty-two of those who received the placebo and 34 of those who received the supplements came for three months follow-up and agreed to give a blood sample and fill in the infection diary. Corresponding figures for 12 months follow-up were 37 and 48, respectively. Exclusions were due to refusal to respond to the infection diary questionnaire. The Figure 1 details the recruitment and intervention process and 3- and 12-month follow-up. 

### 3.1. Baseline Characteristics

Table 1 shows baseline characteristics of the treatment and placebo group. The 2 groups were comparable on entry into the study with respect to age, adiposity, duration and treatment of diabetes and other clinical and biomedical measures. 

**Table 1:** Baseline characteristics placebo and supplement group (mean SD, unless stated otherwise).

		Placebo (*n* = 50)	Supplement (*n* = 50)	*p-*value
Age (years)		51.2 (12)	51.1 (12)	0.973
Male: female		18:32	23:27	0.312
Level of education, *n* (%)	≤High school	38 (76%)	41 (85. %)	
	>High school	12 (24%)	7 (14%)	0.309
Smoking (*n*)		2	6	
Duration of diabetes (years)		2.0 (0.9)	2.2 (0.9)	0.258
Previous hypertension (*n*)		34	28	0.219
Previous IHD (*n*)		8	7	0.781
High cholesterol (*n*)		36	38	0.650
Number of drugs/patient		2.2	2.3	0.849
Treatment of diabetes	Diet	2	2	
	Tablet	36	28	
	Metformin	22	16	
	Metformin + glimepiride	12	8	
	Metformin + rosiglitazone	2	3	
	Metformin + gliclazide	0	1	
	Insulin	9	18	
	Both (metformin + insulin)	3	2	0.059 *
BMI		32.2 (6)	31.4 (7)	0.538
Waist circumference (cm)		105 (14)	102 (12)	0.184
Systolic BP (mm Hg)		134 (16)	137 (19)	0.302
Diastolic BP (mm Hg)		78 (11)	81 (10)	0.147
HbAic (%)		8.1 (2.2)	8.0 (1.9)	0.802
Total cholesterol (mmol/L)		4.7 (1.2)	4.4 (0.8)	0.081
Triglycerides (mmol/L)		1.44 (1.3)	1.49 (1.7)	0.851

* p-value for between groups difference using Kruskal-Wallis one-way analysis of variance.

Although there were some differences in some baseline characteristics, such as gender, smoking, previous hypertension and total cholesterol level, these differences did not reach statistical significance. Ninety diabetic patients out of 100 were either overweight (BMI 25–29.9), (*n* = 30) or obese (BMI ≥ 30), (*n* = 60). *Supplementation*: the median (Q1–Q3) number of tablets (maximum 90 tablets) taken by the supplement and placebo group were 90 (70–90) and 85 (70–90) tablets, respectively. Vitamin supplementation significantly increased plasma vitamin E and serum folate andreduced total plasma homocysteine levels and some of the inflammatory markers in the intervention groups compared with the placebo group (Table 2) [14].

**Table 2:** Baseline and three months plasma antioxidants and inflammatory markers in the intervention group compared with the placebo group (mean SD).

		Placebo (*n* = 50)	Supplement (*n* = 50)	*p*-value *
Vitamin C (mg/L)	Baseline	23.8 (16.8)	33.00 (20.1)	
	3 months	19.5 (12.1)	18.9 (12.8)	0.913
Vitamin E (mg/L)	baseline	7.3 (4.5)	8.6 (3.2)	
	3 months	7.6 (4.6)	11.4 (4.5)	0.006
Folate (nmol/L)	baseline	18.2 (8.9)	18.95 ((8.1)	
	3 months	18.7 (8.6)	32.4 (11.9)	0.001
B12 (pmol/L)	Baseline	236 (103)	179 (93)	
	3 months	227 (99)	252 (191)	0.001
Homocysteine (mmol/L)	Baseline	10.3 (3.2)	12.7 (4.5)	
	3 months	10.7 (3.3)	11.5 (3.3)	0.657
IL6 (pg/mL)	Baseline	3.42 (2.22)	2.49 (1.32)	
	3 months	5.40 (2.53)	3.35 (1.99)	0.023
TNFα (pg/mL)	baseline	1.26 (1.63)	1.66 (2.24)	
	3 months	1.15 (0.8)	0.96 (0.21)	0.204
CRP (mg/L)	Baseline	11.6 (8.9)	10.1 (8.6)	
	3 months	15.1 (15.9)	8.4 (3.4)	0.205

* Three months values were regressed on baseline values and intervention (placebo 1/supplement 2).

### 3.2. Effect of Supplement on Infections

Table 3, Table 4 show the frequencies of infections in the treatment group compared with the placebo group. The total number of reported infections at three months of follow-up in the treatment group was 9/34 subject (27%), compared with 15/42 (36%) in the placebo group (*p* = 0.623) (Table 3). At 12 months, the number of reported infections in the treatment group was 25/37 (67.5%), compared with 27/48 (56.3%) in the placebo group (*p* = 0.488) (Table 4).

**Table 3:** Frequencies of infections over three months in diabetic subjects of treatment and placebo groups (*n* %).

Type of infection	Placebo (*n* = 42)	Treatment (*n* = 34)	*p*-value
Cold	2 (4.7%)	0	
Flu	2 (4.7%)	0	
Sore throats	1 (2.4%)	1 (2.9%)	
Bronchitis	0	1 (2.9%)	
Urinary tract infection	0	1 (2.9%)	
Gastro enteritis (diarrhea and vomiting)	1 (2.4%)	0	
Ear infection	1 (2.4%)	1 (2.9%)	
Other	8 (19%)	5 (14.7%)	
Number of infections per subject	0.36	0.26	0.623

*p* ≤ 0.05 significant.

**Table 4:** Frequencies of infections over 12 months in diabetic subjects of treatment and placebo groups (*n* %).

Type of infection	Placebo (*n* = 48)	Treatment (*n* = 37)	*p*-value
Cold	3 (6.25%)	4 (10.8%)	
Flu	4 (8.3%)	5 (13.5%)	
Sore throats	4 (8.3%)	3 (8.1%)	
Bronchitis	2 (4.2%)	1 (2.7%)	
Urinary tract infection	3 (6.25%)	2 (5.4%)	
Gastro enteritis (diarrhea and vomiting)	0	1 (2.7%)	
Ear infection	2 (4.2%)	1 (2.7%)	
Other	9 (18.8%)	8 (21.7%)	
Number of infections per subject	0.56	0.67	0.488

*p* ≤ 0.05 significant.

### 3.3. Food Intake

Table 5 shows fruits and vegetables intake of the study population at three and 12 months follow-up. Although the intake was reasonably high at three months and increased further at 12 months, there was no significant difference between the two groups, except for the tinned or dried fruits at three months (*p* = 0.000). 

**Table 5:** Food diary in diabetic subjects on treatment and placebo (mean SD).

Variable	Placebo *n* = 43 number/week		Treatment *n* = 32 number/week		*p*-value 3 months	*p*-value 12 months
	3 months	12 months	3 months	12 months		
Apples and pears	3.2 (2.8)	5.5 (2)	2.8 (2.3)	5.5 (1.8)	0.500	0.900
Oranges and bananas	4 (2.6)	5.4 (1.9)	3.8 (2.4)	5.3 (1.6)	0.600	0.900
Tinned or dried fruit, fruit in syrup or juice	1.8 (2.4)	4.2 (2.4)	4 (2.6)	5.2 (2)	0.000	0.090
Fruit (fresh or from a carton)	3.1 (2.6)	3.9 (2.8)	3 (2.6)	3.5 (2.5)	0.800	0.600
Green vegetables, salad, cabbage, spinach	5.6 (2)	6 (0.9)	5.4 (2)	5 (2)	0.700	0.080
Potatoes, mashed, boiled, baked, chips	1.9 (1.9)	3.9 (2.4)	1.6 (1.8)	3.6 (2.6)	0.500	0.700
Vegetables in soup, stews, ready meals, *etc*.	2.6 (2.3)	5 (2)	3.2 (2.3)	5 (1.8)	0.300	0.900
Other vegetables, peas, carrots, onions, tomatoes, *etc*.	4.1 (2.6)	5 (1.4)	4.6 (2)	5.4 (0.9)	0.400	0.500
Average number of fruits and vegetable per day	3.8	5.6	2.1	4.7		

*p* ≤ 0.05 significant.

### 3.4. Exercise and Physical Activity

Table 6, Table 7 reveal the three and 12 months physical activity of the study population. The majority of the diabetic patients in this study reported very low levels of physical activity during work and leisure times. For example, only two patients had a very active occupation and two very active leisure times at three months. Corresponding figures for 12 months were one with a very active occupation and one patient active at leisure time. When patients asked how often they are physically active for at least 20 min where they become out of breath and sweat, the majority of patients answered less than once a week. This sedentary lifestyle was accompanied by a high prevalence of overweight and obesity of up to 90% in the study population and a further increase in body weight—mean body weight at baseline 82.2 kg increased to 86.9 at three months of follow up.

**Table 6:** Exercise diary in diabetic subjects on treatments and placebo at three months.

Variable		Placebo	Treatment	*p*-value
		(*n* = 43)	(*n* = 32)	
		Number (%)	Number (%)	
How physically active is your occupation	not very active	22 (51%)	12 (37.5%)	0.291
	moderately active	18 (42%)	18 (56%)	
	very active	2 (4.6%)	0	
	not working	1 (2.3%)	2 (6%)	
How physically active is your leisure time	not very active	16 (37%)	13 (40%)	0.517
	moderately active	24 (55.8%)	19 (59%)	
	very active	2 (4.6%)	0	
How many hours per week do you spend doing housework	Mean	7	9	0.529
	SD	(5.1)	(10.6)	
How often are you physically active for at least 20 min, where you become out of breath and sweat	<1/week	26 (60%)	24 (75%)	0.402
	1–2/week	8 (18.6%)	7 (22%)	
	3–4/week	4 (9.3)	0	
	5–6/week	1 (2.3%)	0	
	7–8/week	1 (2.3%)	0	
	>8/week	0	0	
How many hours per day do you usually spend in bed (this includes time spent reading, watching television, sleeping)	<1 h	5 (11.6%)	3 (9%)	0.824
	1–2 h	7 (16%)	5 (16%)	
	3–4 h	2 (4.6%)	0	
	5–6 h	9 (21%)	8 (25%)	
	7–8 h	13 (30%)	13 (40%)	
	>8 h	5 (11.6%)	2 (6%)	

*p* ≤ 0.05 significant.

**Table 7:** Exercise diary in diabetic subjects on supplements and placebo at 12 months.

Variable		placebo	Treatment	*p*-value
		(*n* = 31)	(*n* = 31)	
		Number (%)	Number (%)	
How physically active is your occupation	not very active	18 (58%)	21 (68%)	0.416
	moderately active	6 (19%)	6 (19%)	
	very active	0	1 (3%)	
	not working	7 (23%)	3 (10%)	
How physically active is your leisure time	not very active	24 (75%)	20 (65%)	0.372
	moderately active	7 (23%)	10 (32%)	
	very active	0	1 (3%)	
How many hours per week do you spend doing housework	Mean	15.6	12	0.126
	(SD)	(7.9)	(7.1)	
How often are you physically active for at least 20 min, where you become out of breath and sweat	<1/week	15 (48%)	16 (52%)	0.291
	1–2/week	7 (23%)	5 (16%)	
	3–4/week	1 (3%)	5 (16%)	
	5–6/week	1 (3%)	2 (6%)	
	7–8/week	0	0	
	>8/week	7 (23%)	3 (10%)	
How many hours per day do you usually spend in bed (this includes times spent reading, watching television, sleeping)	<1 h	0	0	0.034
	1–2 h	0	0	
	3–4 h	1 (3%)	1 (3%)	
	5–6 h	3 (10%)	8 (26%)	
	7–8 h	9 (29%)	16 (52%)	
	>8 h	18 (58%)	6 (19%)	

*p* ≤ 0.05 significant.

## 4. Discussion

The main findings of this study were that multivitamin supplements improved vitamin blood concentrations and homocysteine and some inflammatory markers. The number of infection per subject reported by the treatment group after three months of supplements was less than that reported by the placebo group; however, this difference was not statistically significant. At 12 months, more infections were reported by the treatment group compared with the placebo group. Although the difference in the number of reported infection at three months might be associated with the vitamin supplementation, the lack of statistical significance could be related to the low dose of the vitamins used or the short period of supplementation or the high intake of fruits and vegetables or a combination of all three factors. We also found very low levels of physical activity among our study population.

The association between diabetes and bacterial infection has been recognized for many years [2,3]. A prospective study of adult inpatients reported that a higher incidence of bacteremia, mainly of urinary source, community-acquired and due to *E. coli* was found in the diabetic patients compared to non-diabetics [15]. Diabetes mellitus may also contribute to surgical wound infection. In another study, the rate of wound infection in patients who had total hip replacement surgeries was 11% in diabetic patients, compared with 2% in non-diabetics patients of similar age [16]. Not all studies have found increased risk of surgical wound infection in diabetic subjects [17,18]. 

The mechanisms leading to the diabetes-related decline in innate and adaptive immunity are poorly understood, and currently, there are no effective strategies for preventing immune senescence in diabetic patients. However, there is good evidence that improving nutritional status might attenuate the decline in immune function in other high risk groups [19,20,21]. For example, randomized control trials suggest that single-nutrient supplements of vitamin E [19], vitamin A [22], zinc [23] and selenium [24] enhance aspects of immune function in older people. Similarly, multi-nutrient supplementation has been reported to improve immune status in older adults [25]. Vitamin C may also play a role as an antioxidant in protecting the immune system against damage from free radicals released during the inflammation process [26]. B vitamins including folic acid play a role in reducing the levels of homocysteine, which showed an association with immunity in the elderly [27]. 

Although not all diabetic patients have micronutrient deficiency, some deficiencies have been identified in a subgroup of patients. For example, serum ascorbic acid and group B vitamin concentrations may be low in diabetic patients [9]. Barringer and colleagues have reported that multivitamin and mineral supplements significantly reduced self-reported infections in 130 healthy people ages 45 to 64 years or older, with most of the beneficial effect seen in undernourished people with diabetes [28]. Although these studies provide evidence of an association between micronutrients and immune function, the majority of them have used micronutrient doses in excess of recommended requirements and the results cannot therefore be readily translated into dietary guidelines. Few studies have attempted to use foods or doses of nutrients that are realistically achievable through diet to modulate immune status [29]. Such studies are important, as they demonstrate the effects of individual components of the diet, but do not consider the benefits of a mixed diet, nor are they designed to provide a method of sustainable dietary change amenable to the majority of the target population. 

Although we found a high level of fruits and vegetables intake in our study sample, up to 90% of these subjects were either overweight or obese, and their body weight increased further during three months of follow-up. The high rate of overweight and obesity is also associated with a sedentary life style. These changes in lifestyle are almost certainly leading to the growing epidemic of overweight/obesity, type 2 diabetes and other related co-morbidities in the UAE society and other similar population and are therefore in need of urgent public health attention. Although obesity as a risk factor for several morbid conditions is well accepted, its contribution to infection has not been sufficiently studied. Some studies have suggested that infections of several body systems are more frequent in obese people than those of normal weight [30].

*Strengths and weaknesses*: It is possible that if the vitamin doses were higher, the supplement period was longer or the sample size was larger, we might have seen bigger differences in the rate of infections between the supplement and the placebo group. Although compliance with the trial medications was very good and supplements increased vitamin levels significantly in the blood, nearly one quarter of our subject sample refused or were unable to report infections at follow up. In this study, we have relied on the patient self-reported incidence of infections. This might have introduced a margin of error in their reporting of the true infection rate. It is possible that we could have obtained more accurate and reliable information on infection rate if another method of collecting information was used, such as reviewing patient’s hospital records, tracking clinic records where the participants had visited and obtained treatment or simply having patients call for every single episode of infection he/she experienced, or even conducting more regular visits or calls to check the condition of the patient, but this would require more resources and is very time-consuming. Another important weakness is that we did not measure any biological marker of cellular or humeral immunity. 

## 5. Conclusions

In conclusion, we found that dietary supplements improved the concentrations of the blood vitamin levels over the three months; however, this improvement did not translate into low risk of infections over the 12 months follow-up period. A larger clinical trial is needed to find out whether a higher daily dose of vitamins and/or longer duration of supplements in diabetic and non-diabetic patients at higher risk of infection is likely to lead to a clinical benefit. If vitamin supplements are found to reduce the rate of infections in diabetics and other similar group of patients, wider implementation of this strategy could have significant health and economic implications on diabetic patients in the UAE and elsewhere.
